# Supplementary material for: Diagnostic Value of EUS-FNA in the Differential Diagnosis of Esophageal Strictures Lacking Typical Malignant Features
Source: Diagnostics (Basel). 2025 Sep 26;15(19):2470. doi: 10.3390/diagnostics15192470 (PMC12524196; doi:10.3390/diagnostics15192470)
Supplement: Supplementary file 1 [file diagnostics-15-02470-s001.zip › diagnostics-3839050-supplementary.pdf]

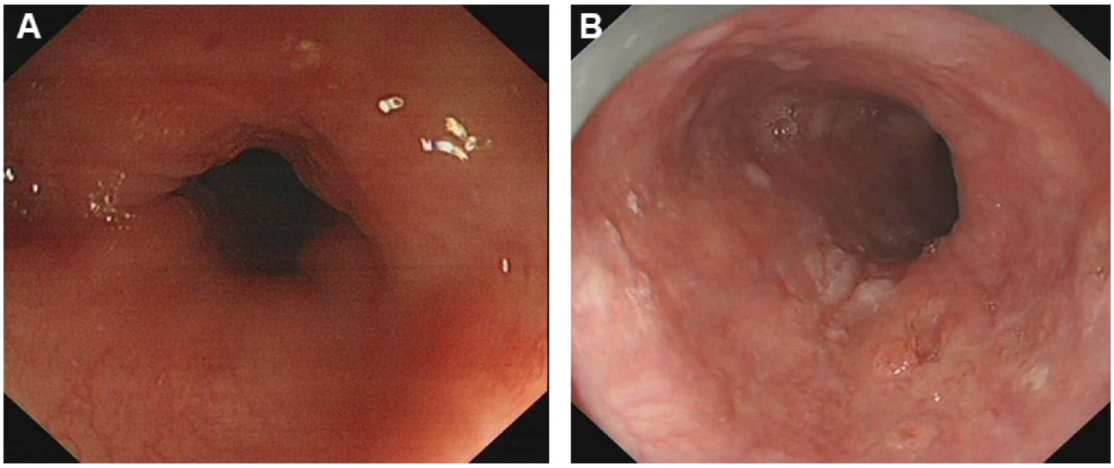

Figure S1. (A) Endoscopic image from Patient #12 (diagnosed with esophageal squamous cell carcinoma) showing coarse mucosa with small nodular bulges; (B) Endoscopic image from Patient #9 (diagnosed with esophageal squamous cell carcinoma) showing smooth esophageal mucosa with no significant ulcers or erosions.
